# Age and gender related neuromuscular changes in trunk flexion-extension

**DOI:** 10.1186/1743-0003-12-3

**Published:** 2015-01-07

**Authors:** Thomas Kienbacher, Birgit Paul, Richard Habenicht, Christian Starek, Markus Wolf, Josef Kollmitzer, Patrick Mair, Gerold Ebenbichler

**Affiliations:** Karl-Landsteiner-Institute for outpatient rehabilitation research, Vienna, Austria; Department of physical medicine and rehabilitation, Medical University of Vienna, Vienna, Austria; Technical school of engineering, Vienna, Austria; University of biomedical engineering, Vienna, Austria; Department of psychology, Harvard University, Cambridge, MA USA

**Keywords:** Lumbar extensor muscles, Trunk flexion-extension, Electromyography, Age, Gender, Kinematics

## Abstract

**Background:**

The root mean square surface electromyographic activity of lumbar extensor muscles during dynamic trunk flexion and extension from a standing position and task specific spine ranges of motion objectively assess muscle function in healthy young and middle age individuals. However, literature on neuromuscular activation and associated spine and hip kinematics in older individuals is sparse. This cross sectional study sought to examine the sex and age (<40 versus >60 years) related differences in the neuromuscular activation profiles of the lumbar extensors and the related spine and hip kinematics from healthy individuals during a standardized trunk flexion-extension task.

**Methods:**

Twenty five older (13 females, 60–90 years) and 24 younger (12 females, 18–40 years) healthy individuals performed trunk flexion-extension testing by holding static positions at half-flexion way and full range of motion between standing and maximum trunk flexion. The associated lumbar extensor muscle activity was derived from measurements at standing, half, and maximum flexion positions. The range of motion at the hip and lumbar spine was recorded using 3d accelerometers attached to the skin overlying the multifidus and semispinalis thoracis muscles lateral to the L5 and T4 spinous processes, respectively. Statistical calculations were performed using a permutation ANOVA with bootstrap confidence intervals.

**Results:**

The muscle activity in the half related to the maximum flexion position (half flexion relaxation ratio) was significantly smaller in older males when compared with younger males. Moreover, measurements revealed smaller activity changes from standing to the half and from half to the maximum flexion position in older compared to younger individuals. Older males displayed smaller gross trunk range of motion from standing to maximum flexion than any other group.

**Conclusions:**

Gender and normal aging significantly affect both the activation patterns of the lumbar extensor muscles and the kinematics of the trunk during a standardized trunk flexion-extension task. Measurement results from healthy young and middle age individuals should not be used for the assessment of individuals older than 60 years of age.

**Electronic supplementary material:**

The online version of this article (doi:10.1186/1743-0003-12-3) contains supplementary material, which is available to authorized users.

## Background

Flexion and extension of the trunk from a standing position is governed by a complex system involving sensory (ligaments, golgi tendon organs, receptors in capsules), neural, active (muscle), and passive (bones, discs, and fascia) structures. Spine flexion normally dominates the first two thirds of gross trunk flexion while lumbar extensor muscle activity augments up to a peak [1–3]. When the posterior ligaments (supra- and intraspinosus, posterior longitudinal) become completely stretched further flexion is led by the hips. At this point the ligamento-muscular reflex normally allows relaxation of lumbar extensor muscles [2, 4–6]. Maximum lumbar extensor muscle root mean square (RMS) surface electromyographic (SEMG) activity, it`s ratio to the respective muscle activity in maximum flexion position, and the task specific range of motion (ROM) of the spine have all been used in past studies to reliably differentiate normal from abnormal findings in individuals younger than 55 years of age undergoing dynamic trunk flexion-extension testing. Moreover, it was recommended that such measurement results are used to assess treatment outcome in low back pain patients [7]. However, these findings need to be confirmed for older healthy populations [4, 5, 7–9].

Reduction of lumbar flexion-extension range of motion starts at around 50 years of age, decreasing further after the age of 60 [10, 11]. Moreover, a reduction in the fraction of water within the vertebral disc and the surrounding tissues causes stiffness of the spine [12]. The deterioration of the viscoelastic properties of dorsal ligaments decreases their effectiveness as sensory organs [2]. Accordingly back muscle reflex latency of older individuals has been found to be delayed in response to loading of the spine along with less activity among trunk muscles in older adults performing functional tasks [13]. Such neuromuscular activation changes become dominant starting at 50 years of age when restrictions of range of motion start to manifest [14–17]. Thus one can speculate that age associated changes likely have an impact on measurement results of kinematics and neuromuscular activation of lumbar extensors in trunk flexion-extension testing.

The authors of this study identified only one study that established SEMG activation profiles of lumbar extensor muscles and the related trunk kinematics for a healthy population older than 60 years of age [18]. In this study, the small group of 12 older volunteers revealed persisting higher muscle activities throughout the task with less increment towards their peaks compared to younger participants. Despite the authors` attempt to pace trials, when compared to young volunteers, older individuals had difficulty complying with the dynamic test protocol. Their movement velocities varied during relevant parts of the task affecting the respective SEMG measurement results [18, 19]. Moreover, the signal derived from bipolar surface electrodes may have been modulated differently depending on volunteers` age related restriction of the spinal range of motion when electrodes moved further apart and closer together following stretching and shrinking of the skin resulting from flexion and extension of the trunk. Furthermore this study did not evaluate the differences between sexes in the age associated lumbar range of motion restrictions [11].

EMG assessment using constant inter-electrode distance could help to alleviate the effect of different maximal range of motion especially when comparing older with younger individuals. Moreover testing in static key positions could be easier to perform by an older population, thereby ruling out the movement velocity factor [20].

The aims of this study were to investigate the sex and age (<40 versus >60 years of age) related differences in lumbar extensor muscle activity recorded at half and maximum trunk flexion (half flexion relaxation ratio, HFR). Secondary aims of the study were to compare the normalized lumbar extensor muscle activity among standing, half, and maximum trunk flexion positions, the relative activity changes between these positions and the task specific hip, lumbar, and gross trunk ranges of motion.

## Methods

### Participants

A total of 25 healthy older (13 females, 60–90 years) and 24 healthy younger (12 females, 18–40 years) volunteers comprised of hospital staff, personal contacts of the examiners, and community dwelling individuals were enrolled in this cross sectional study. Participants were also recruited through advertising presentations in leisure time institutions for older persons and companies. Physicians specialized in Physical and Rehabilitation Medicine (PRM) screened all volunteers. Individuals were eligible for the study if they were free of co-morbidities (mild diabetes, well controlled hypertension, mild osteoarthritis of lower limb weightbearing joints were included), had no history of spine surgery nor any kind of specific spine disease, no history of back pain or a maximum of five mild (visual analogue scale < 30) episodes of back pain and/or referring pain to the head, arms, or legs within the past year, did not seek healthcare advice for back pain within the last year, had no major general health problems that would interfere with testing, were free from any functional limitations (reported independent walking distance exceeded 800 m, timed up-and-go test less than 10 s [21], Tandem-stand exceeded 10 s [22], stand-up test less than 10 s [23]), were independent in their activities of daily life, exhibited normal physical activity levels but did not perform sports on a competitive level more than 2 times per week, had no clinical findings indicative of neuropathological conditions or structural impairments, and a body mass index lower than 30 kg/m^2^.

### Schedule of tests

The basic steps were as follows: 1) Questionnaires that assessed demographic variables, the SF-36 health survey [24] for physical and mental functioning, and the International Physical Activity Questionnaire (IPAQ) [25] for physical activity level were filled out on tablets by the volunteers under supervision of the examiners, 2) muscle warm-up followed by maximum isometric back extension (100% MVC) strength testing, 3) a 30 minute pause for recovery, 4) trunk flexion-extension testing, and 5) 80% MVC back extensor muscle SEMG recording for normalization.

### Instrumentation (equipment and tests)

#### Maximum (100% MVC) back extension dynamometer

Maximum isometric back extensor muscle strength was measured using a specially designed device (F110 extension; DAVID® Health Solutions Ltd, Helsinki, Fi), as described in [26]. In short, the device consists of a “hip fixation mechanism” that is comprised of five components: footplates adjustable to lower leg length, knee pads adjustable to thigh length, a pelvic belt, a seat adjustable for height, and a dorsal back pad. According to the manufacturer`s recommendations, participants were seated with their knees fixed and flexed at 90°-95° and their trunks flexed forward at 30° relative to the vertical. Strength gauges attached to the test devices measured the trunk extension torque in Nm and results were displayed in real time on the monitor (EVE®) attached to the device for visual feedback.

#### 80% MVC back extension dynamometer

In order to obtain undisturbed RMS SEMG recording from the back extensor muscles, participants performed the back extension test at 80% of maximum back extension strength on the Total Trunk (Technogym®, Gambettola, Italy) device. This back extension device consists of a hip fixation mechanism which is similar to that of the DAVID® device but includes a dorsal sacral pad instead of a back pad thus avoiding pressure on the sensors while testing. Like the David° device, the Total Trunk has footplates that are adjustable to lower leg length, knee pads that adjust to thigh length, a pelvic belt, and a seat adjustable for height.

#### RMS SEMG and landmarks

Electromyographic signals and landmark position data were recorded using active double parallel-bar electrode sensors with integrated triaxial accelerometric sensors (Model Trigno, DelSys Inc®, Boston, MA, USA). Reference electrodes are built into the Trigno SEMG system, with constant interelectrode distance of 10 millimeters [27]. The SEMG signals were acquired using four active electrodes that provided a total effective gain of 909 V/V ± 5%, a bandwidth of 20 – 450 Hz, and a baseline noise < 0.75 μV (RMS). The SEMG signals were sampled at 2000 Hz with a resolution of 16 bits using EMG Works® acquisition software. The triaxial accelerometers acquired preamplified signals with a dynamic range of ± 1.5 g, a maximum resolution of 0.016 g/bit and a bandwidth of dc-50 Hz. The accelerometer signals were sampled at 160 Hz with a resolution of 8 bits and EMG Works® acquisition software. After the skin at the electrode sites had been abraded with alcohol and, shaved if necessary, the electrodes were positioned bilaterally over the multifidi muscles at L5 (line between iliac crests, 2–3 cm bilateral und distal from median) and the semispinalis thoracis muscles at T4 (four vertebral segments caudal from C7 and 2–3 cm bilateral). The electrodes were positioned according to the muscle fiber directions and the positioning recommended by the SENIAM project [28] and Larivière et al. [29] for L5 level. All sensors were secured to the skin by a double sided adhesive interface.

### Test procedures

Data were collected between June 2011 and March 2012. The data collection was carried out in accordance with the directives given in the Declaration of Helsinki and has been recognized by the Vienna ethical committee. Volunteers received oral and written information about the study and signed an informed consent form. All tests were performed in a constant order mid-morning to avoid effects of diurnal changes of performance. Detailed written, verbal, and demonstrative instructions of the required tasks were given to all volunteers until they had no further questions.

#### Maximum (100% MVC) back extension test

Volunteers performed at least three slow, sub-maximum dynamic warm-up trials within the full range of trunk motion at low loads and practiced one or two isometric test contractions at submaximum loads using feedback on the visual display. Thereafter, they generated maximum isometric contractions by gradually increasing the torque up to their maximum capacity within the first 2–3 s of each contraction. The entire strength evaluation was performed under supervision of the experienced examiner. The best value obtained out of two attempts was stored. If test values varied by more than 10%, or if the maximum torque was achieved later than 3 s after the onset of the contraction, then further trials were permitted until a consistent maximum was achieved. Intervals between maximum test repetitions were a minimum of 15 s. Verbal instructions and encouragement were standardized according to the recommendations of a clinical psychologist.

#### 80% MVC back extension test

Volunteers were seated on the Total Trunk® device using the same positioning variables that were used for the DAVID® device. After the volunteers had been secured in the device and all restraining mechanisms and lever arm attachments had been adjusted, the lever arm was loaded with 80% of the maximum load generated on the DAVID® device. The 80% maximum voluntary contraction (MVC) load in kg was calculated from the best maximum trunk extension moment (Nm) obtained from the DAVID® device. This was obtained by the mathematical product of the moment as recorded by the load cell of the dynamometer and the moment arm defined by the perpendicular distance between the L5/S1 joint and the load cell of the back restraint. With support from the tester, the participants moved their trunk into a 30° anteflexed (relative to the vertical) position. From this position participants were instructed to be ready to maintain the position constant for 30 s. The first stable phase of 4 s of the sustained contraction which occurred within the first 10 s after the starting point was recorded. Volunteers were allowed to truncate their muscle contractions immediately thereafter.

#### Modified trunk flexion-extension test after Watson et al. [4]

Sensors were positioned for continuous recording of lumbar extensor muscle SEMG and landmark position data using the embedded accelerometers. Volunteers practiced up to five warm-up trials for verification of proper accelerometer function until they felt comfortable with the task (no pain or vertigo) but not fatigued [30–32] and were able to perform at the given velocity that equated 2 s for each movement phase between positions (plus periods for holding static positions) for a full flexion and re-extension paced by a metronome. Special emphasis was put on maintaining a slow and continuous movement velocity without abrupt contractions or side bending and on stops at 50% trunk flexion relative to the task specific maximum trunk flexion. Volunteers were positioned in a relaxed standing position with their feet shoulder width apart, knees extended, arms hanging freely to their sides and looking straight ahead. Head position relative to the cervical spine was kept constant during testing as different positions could have profound effect on lumbar extensor muscle activity [33]. Correct test performance was controlled by clinical observation, continuous reading of the raw amplitude SEM signal, and online data inspection by the examiners. Adjustments were made whenever necessary. Volunteers remained in standing position until the required 4 s stable accelerometer live stream signal without movement artefacts occurred. They were then told to slowly flex their trunk forward at the designated and practiced movement velocity until the examiner asked them to stop half way between standing and maximum trunk flexion (i.e. half position at 50% of task specific trunk flexion) and to remain in this position for 10 s. When the signal became stable for 4 s in this position and when 10 s were over, volunteers were asked to slowly further flex their trunk until the point where they felt maximum relaxation of their lumbar extensor muscles while focusing on “passively hanging in their dorsal ligaments and passive structures and relaxing back extensor muscles as much as possible” (i.e. maximum flexion position) and to remain there for another 10 s. When the 4 s of stable live stream had occurred and the 10 s were over volunteers were asked to re-extend their trunk back to the half position (extension phase) at the same velocity. After another 4 s stable phase within a 10 s period volunteers were asked to return to the standing position at the standardized velocity, and to remain there for another 10 s to record accelerometric data accordingly. The procedure was repeated twice without pausing.

### Data processing

The raw SEMG signals were initially filtered with a fifth-order high-pass Butterworth filter with zero phase lag (cutoff frequency 45 Hz) to attenuate artefacts. The envelope of the SEMG signal was then obtained using a 12 Hz low-pass filter (FIR implemented using a 201-coefficient Hamming window) and down sampled by a factor of 10. The RMS values were obtained as the mean of the first 4 s window of stable data that usually occurred 2 to 3 s after the onset of a sustained back extension and were automatically identified by the angular bending information from the 3d-accelerometer signals. Such procedure allowed EMG analysis from stable contraction positions [34]. Complete task raw RMS SEMG signals and accelerometer position data were monitored in real time by the tester recording movement velocity and identifying motion artefacts. Remaining artefacts were eliminated according to amplitude and frequency detection with zero paddings and visual inspection.

Parallel 3d-acceleration measures were taken at levels for lumbar extensor muscle RMS SEMG activity and for L5 and T4 landmark position data and down sampled by a factor of 10. Sagittal angular displacement was calculated by a geometrical procedure using direction of gravity as reference. For calculation of individual angles, the acceleration data of the electrodes from every position was used. A simple angle calculation with two vectors was performed as described in


where α is the angle, a is the acceleration vector in cranio-sacral direction and g is the acceleration due to gravity. Gravity/position data and RMS EMG data from the standing (pre- and post-flexion), the half-flexion position (during flexion and extension phase) from the right and left sides and from the trial repetitions were similar indicating that no relevant trunk rotation occurred. 3d-accelerometer position data and RMS SEMG data from standing pre- and postflexion, from half position during flexion and extension phase, from right and left side, and from the trial repetitions were similar and thus averaged for further calculation. All RMS SEMG data were normalized to 80% MVC measurement results of the same volunteer.

For calculation of the task specific hip, lumbar, and gross trunk ROM, the following calculations were performed:

- hip ROM: mean position L5 in maximum flexion minus mean position L5 in standing.
- lumbar ROM: mean position in maximum flexion (difference T4 – L5) minus mean position in standing (difference T4 – L5).
- gross trunk ROM: mean position T4 in maximum flexion minus mean position T4 in standing.

The main outcome was the half flexion relaxation ratio (HFR) calculated from the average of the four RMS SEMG lumbar extensor muscle amplitudes in the half devided by the respective average of the two amplitudes in maximum flexion position that had been derived from the respective RMS EMG recording levels of the 2 test repetitions.

Secondary outcome variables aimed at generating a more in-depth understanding of age and gender related changes of the HFR and were:

- mean normalized extensor muscle RMS SEMG amplitude during standing, half, and maximum flexion position,
- changes of the mean normalized lumbar extensor muscle RMS SEMG amplitudes between the positions,
- hip, lumbar, and gross trunk ROM for the total task,
- % of hip and % of lumbar motion contributing to gross trunk ROM of the total task, and
- hip, lumbar, and gross trunk ROM between standing and the half flexed position.

### Statistical analysis

All statistical analyses were performed in the R environment for statistical computing® [35]. The inference part of the analysis consisted of several 2x2 ANOVAs on the main outcome variables. For these outcomes normality checks were performed (histogram, Q-Q-plots, and Shapiro-Wilks tests). Some of the outcomes violated the normality assumption because the corresponding frequency distributions were skewed. Therefore, a permutation ANOVA using the lmPerm package [36] was applied. For the subgroup means bootstrap confidence intervals were used. Results were inspected graphically by interaction plots (plots not shown). The significance level was set at α = 0.05.

## Results

A total of 49 healthy volunteers completed the experiments, 25 of them were aged 60–90 years (13 females) and 24 of them 18–40 years (12 females). Demographic variables, results of SF-36 subscores, and IPAQ are provided in Table 1.

**Table 1:** **Demographics, SF-36, and IPAQ**

	<40 (n:24)		>60 (n:25)		Men (n:24)		Women (n:25)		w| < 40 (n:12)		m| < 40 (n:12)		w| > =60 (n:13)		m| > =60 (n:12)	
	Mean	SD	Mean	SD	Mean	SD	Mean	SD	Mean	SD	Mean	SD	Mean	SD	Mean	SD
**Demographics**																
Age	25.5	06.3	70.9	07.4	48.8	23.6	48.5	24.8	24.2	06.7	26.8	05.9	71.0	06.7	70.8	08.3
Heigth^1^	174.3	09.3	168.6	10.4	179.5	06.4	163.6	06.4	167.0	05.2	181.6	06.2	160.5	06.1	177.3	06.1
Weight^2^	71.90	11.33	72.02	13.78	80.05	10.42	64.19	08.97	65.71	08.91	78.10	10.26	62.79	09.15	82.01	10.66
BMI^3^	23.64	03.03	25.15	02.87	24.86	02.95	23.97	03.08	23.56	03.10	23.71	03.09	24.35	03.14	26.01	02.39
**SF-36**																
PCSS^4^	44.93	01.85	43.46	01.99	44.57	02.03	43.81	02.02	44.65	01.86	45.22	01.87	43.03	01.90	43.93	02.05
MCSS^5^	51.88	09.18	56.41	06.97	54.39	06.55	54.00	09.93	50.84	11.67	52.92	06.14	56.92	07.29	55.86	06.88
**IPAQ**																
Total Physical Activity	350.16	446.95	382.43	372.80	443.00	418.70	293.31	389.25	339.67	547.61	360.65	342.89	250.52	154.94	525.35	483.93

^1^in cm ^2^in kg ^3^BMI = Body Mass Index ^4^PCSS = Physical component summary score ^5^MCSS = Mental component summary score.

### Mean RMS SEMG half flexion relaxation ratio (HFR) of lumbar extensor muscles

The ratio recorded at half and maximum trunk flexion (HFR) revealed significantly lower values in older males in comparison to all other subgroups (younger males and both female groups) and in older females compared to younger males (Table 2) meaning that the flexion relaxation phenomenon was less marked in these groups.

**Table 2:** **Normalized RMS SEMG amplitudes, kinematics, and differences**

	<40 (n:24)		>60 (n:25)		Men (n:24)		Women (n:25)		Age			Gender			Age x gender		
	Mean	SD	Mean	SD	Mean	SD	Mean	SD	RSS	p-value		RSS	p-value		RSS	p-value	
**RMS SEMG amplitude (in percent):**																	
standing/80% MVC	29.62	24.38	36.24	22.96	34.30	22.22	31.75	25.36	127.7	0.4296	ns	446.6	0.2444	ns	688.3	0.2776	ns
half/80% MVC	61.10	38.47	53.41	24.94	62.39	27.75	52.17	35.77	1151.1	0.2078	ns	1836.2	0.1747	ns	2983.5	0.0669	ns
max flexion/80% MVC	35.73	35.79	50.35	29.84	47.26	37.58	39.28	29.00	1560.8	0.1851	ns	1640.1	0.0985	ns	5656.1	0.0140	*
**Relative EMG changes (in percent):**																	
standing - half	31.48	22.70	17.16	17.60	28.08	15.66	20.42	25.37	2045.6	0.0166	*	471.7	0.3136	ns	805.3	0.1506	ns
standing - max flexion	06.11	23.37	14.10	17.97	12.96	22.34	07.53	19.63	795.68	0.1460	ns	370.3	0.3004	ns	2398.3	0.0168	*
half - max flexion	25.37	29.03	03.06	09.99	15.13	20.05	12.90	27.81	5392.8	0.0002	*	6.1	0.8431	ns	424.1	0.4013	ns
**Half Flexion Relaxation Ratio (HFR)†:**																	
half/max flexion	02.85	01.98	01.18	00.30	02.41	02.06	01.60	00.93	32.9	<0.001	*	7.3	0.0279	*	13.1	0.0020	*
**Range of Motion (in degrees):**																	
hip (standing - max flexion)	54.89	09.93	59.99	13.98	53.69	13.04	61.15	10.58	278.6	0.1560	ns	627.5	0.0358	*	399.8	0.0908	ns
lumbar (standing - max flexion)	55.11	08.83	39.55	10.92	46.07	15.37	48.23	09.42	3145.9	<0.001	*	43.0	0.5341	ns	613.3	0.0140	*
gross trunk (standing - max flexion)	110.01	10.76	99.54	21.85	99.76	21.13	109.38	13.00	1552.0	0.0066	*	999.2	0.0298	*	2003.4	0.0106	*
hip (standing - half)	27.27	08.89	30.34	08.28	27.70	08.71	29.91	08.61	113.5	0.2983	ns	58.8	0.6604	ns	2.1	0.9412	ns
lumbar (standing - half)	25.33	14.87	25.12	10.43	24.68	11.49	25.77	14.04	0.5	0.9412	ns	14.3	0.8235	ns	434.9	0.1938	ns
gross trunk (standing - half)	52.59	11.95	55.46	12.79	52.37	11.40	55.68	13.23	98.3	0.8431	ns	131.0	0.4737	ns	498.1	0.3875	ns
% hip of gross trunk (standing - max flexion)	54.80	22.92	55.50	12.34	54.16	17.11	56.14	19.57	5.8	0.8824	ns	47.1	0.9216	ns	450.1	0.2903	ns
% lumbar of gross trunk (standing - max flexion)	45.20	22.92	44.50	12.34	45.84	17.11	43.86	19.57	5.8	0.7843	ns	47.1	0.6154	ns	450.1	0.5333	ns

*p < 0.05, ns = non significant, RSS = residual sum of squares.

† Note: In order to keep the data presentation in a uniform manner we provide only the means and SD for all outcome variables. In some cases the normality assumption is violated where frequency distributions are skewed.

### Normalized mean RMS SEMG amplitudes of lumbar extensor muscles and the respective changes between positions

Amplitudes recorded from the standing and half positions were similar in all groups whereas they were higher in maximum flexion when older males were compared to older females. Increments of the amplitudes from standing to the half and the respective decrements from half to the maximum flexion position were significantly smaller in older when compared to younger volunteers. The increments from standing to the maximum flexion were higher in older males when compared to older females or to younger males (Table 2, Additional file 1).

### ROMs during trunk flexion-extension testing

Total task specific hip ranges of motion from standing to the maximum flexion position were lower in males than in females.

The older male group revealed lower task specific lumbar range of motion compared to younger males or younger females. Older females were restricted in their respective range of motion compared to younger males.

The absolute gross trunk ranges of motion between standing and maximum flexion position demonstrated lower values in older males compared to any of the other groups tested but the relative contributions of the hips and the lumbar spine (in %) to gross trunk movement were similar in all groups tested (Table 2, Additional file 1). There were no age or gender specific differences in hip, lumbar, or gross trunk ranges of motion between standing and the half position.

## Discussion

This study sought to comprehensively examine potential age and sex related differences in the lumbar extensor muscle activation pattern as expressed by the HFR and the ranges of motion during trunk flexion and extension. Older volunteers displayed less modulation of lumbar extensor muscle activity in full trunk flexion. Specifically older males revealed lower HFRs, higher muscle activity in the maximum flexion position, and a restriction in both lumbar and gross trunk range of motion. These findings indicate different task specific neuromuscular activation profiles and kinematics that are evident according to age and sex.

Increments of lumbar extensor muscle activity from standing to the half position and the reflectory muscle relaxation thereafter were significantly smaller in older compared to younger individuals. Such low muscle activity modulation in older individuals despite identical biomechanical needs to overcome gravity was surprising. When trunk positions and increments of ROM from standing to the half flexion position do not differ between age groups, activity of lumbar extensor muscles would be expected to increase similarly in younger and older persons. However, RMS-EMG augmented less in the older group. In the older group, however, more motor units would likely be additionally recruited during the half positions relative to standing [37] which should result rather in higher than lower RMS increases. There are a couple of possible explanations for this finding. Recent research detected increased synergistic contribution of the psoas major and quadratus lumborum muscles to the back extension moment in older persons thereby reducing the load to the multifidi muscles [38, 39]. Due to the bigger distance of these muscles to the electrode recording site at L5 their respective activities would be weakly represented in the EMG of the older individuals. Another possible mechanism could be that increased co-activation of deep abdominal muscles in older persons might increase abdominal pressure and thereby reduce the load to back extensors [40–42]. However this would not explain the low activity decrements from the half to the maximum flexion position. A final possible explanation is that aging is accompanied by a loss of type II fibre area [43–45]. Accordingly older individuals have difficulty generating focused and rapid torque (power bursts) against balance disturbances while performing functional tasks [46]. Therefore one could speculate that high level back extensor muscle activity in maximum trunk flexion could be intended to improve postural stability and to protect spine structures from further damage through age induced degeneration [16, 47].

It is important to note that the trunk inclinations in all groups tested were similar in all three test positions (standing, half, and maximum flexion). Furthermore the relative contributions of the hips and lumbar spine (in %) to the total task specific gross trunk movement from standing to maximum flexion were similar in all groups. The half position was chosen equally in all groups at 50% of the task specific gross trunk flexion and thus well before the ligamento-muscular reflex was expected to occur. Accordingly preliminary testing had revealed that more than 90% of volunteers experienced their muscle activity peaks between the half and the maximum flexion position. Moreover with implementation of the half position into a dynamic trunk flexion-extension test protocol the older volunteers could easily comply with test standardization [18].

High muscle activity in maximum flexion position of the older male individuals relative to the respective activity in the half position lead to the lowest HFRs. This group displayed the lowest lumbar motion whereas older females` lumbar range of motion was restricted in comparison to younger males only but not to younger females with a trend towards higher lumbar flexion compared to the older males. The age associated degeneration of discs and surrounding structures and a loss of viscoelastic mechanical properties were demonstrated to correlate with the restriction of flexion-extension range of motion of the spinal segments [12, 48]. Thus both the dominant restriction of lumbar range of motion and the associated stiffness induced loss of proprioceptive ligament input could have contributed to a further deterioration of the ligamento-muscular reflex arc in the older male group. Consequently the HFR and the increments of lumbar muscle amplitude from standing to maximum flexion in older females were rather similar to those from the younger individuals. Neblett et al. [9] postulated that the impaired flexion relaxation reflex and the restricted lumbar range of motion in a group of young and middle age back pain patients were related to previously performed surgery causing extensive damage such as muscle injury, scaring, and degeneration. One possible explanation for our result is that similar underlying processes in these patients and the older healthy male individuals from the current study might be responsible for the changes of the neuromuscular activation pattern.

### Clinical implications

This study revealed, that the specific flexion-extension task is feasible for an older population. Half flexion relaxation ratio (HFR) and the task specific ranges of motion of the spine and hips could be used for the assessment of impaired ligamento-muscular control of the spine. This simple and easily available functional test has a great potential to become a screening tool for detecting neuromuscular alterations in older healthy individuals who may benefit from exercise interventions intended to improve or maintain trunk stability. However further research will need to address whether and how abnormal findings from this test would relate to altered back muscle function and coordination, impaired mobility, limited activities and/or restricted partizipation in older persons. Moreover, the potential use of the HFR as an treatment monitoring tool both in older healthy persons and patients will have to be clarified in the future.

A number of limitations in this study have to be addressed. Data presented are specific for the population tested, the specific trunk flexion-extension task with a testpoint in half position, specific 3d-accelerometers with constant inter-electrode distance, positioning of the devices in L5 (multifidi muscles) and T4 levels, method of data analysis, and the normalization procedure. Comprehensive comparison between measurement results of a dynamic versus a static trunk flexion-extension testing protocol was not performed as this was not part of this study.

Considering volunteers` safety, we chose to record the EMG used for normalization purposes from an 80% rather than a 100% maximum back extension strength test. Undisturbed SEMG recording from the back extensors was feasible only with equipment without a back pad and thus different to that utilized for the 100% MVC testing (David®). This different device (Technogym®), however, would have been disadvantageous for a full 100% MVC testing because it decreases pelvic stabilization and assumingly therefore likely increases the risk of back injury. Based on observations of a linear relationship between the RMS EMG and muscle strength/torque in isometric contractions up to 80% of maximum which becomes more variable if contraction strength further increases we reasonably feel that the procedure in this study would be more than justified. At 80% MVC motor unit recruitment is likely completed in back extensors and firing rates are not expected to additionally increase if static force is augmented higher than 80% MVC. This would be the case particularly if the principles of a hierarchical inverse relationship between the recruitment thresholds and the magnitude of the firing rates with the low threshold motor units exhibiting the highest firing rates applied [49], which has not been shown for the back extensors so far. Moreover, methodical limitations when estimating electromyographic motor unit activity due to a loss of information that is known to occur from overlap of positive and negative phases of motor unit potentials with consecutive partial or complete cancellation of motor unit action potential trains [50] or the variable contribution of agonist (and antagonist) co-contractors [51] may be expected among others to modulate such linear SEMG/force relationship especially in submaximum contractions exceeding 80% MVC.

## Conclusions

Flexion-extension testing with a testpoint in half position is suitable for the assessment of muscular function in an older population. Neuromuscular activation profiles of the lumbar extensors and the related ranges of motion of the spine and hips are specific to age and gender and need to be assessed accordingly. Results from this study should be re-evaluated comparing healthy individuals of different ages with patients suffering from chronic back pain and other pathologic spine conditions.

## Electronic supplementary material

Additional file 1: **Interaction plots of measurement results.** (PDF 210 KB)

## Abbreviations

RMS SEMG: Rout mean square surface electromyography (−ic)
ROM: Range of motion
MVC: Maximum voluntary contraction
HFR: Half flexion relaxation ratio
PRM: Physical medicine and rehabilitation
3d-accelerometer: Three dimensional accelerometer
L5: Lumbar spine level 5
T4: Thoracic spine level 4
SF-36 questionnaire: Short form health survey of 36 questions
s: Second
kg/m^2^: Kilogram per square meter
°: Degree
g: Gravity
dc: Direct current
g/bit: Grams per bit
FIR - Filter: Finite imulse response
V: Volt
*μ*V: Microvolt
mV: Millivolt
Hz: Hertz
%: Percent
*fs*: Frequency.
