# Supplementary material for: Age and gender related neuromuscular changes in trunk flexion-extension
Source: J Neuroeng Rehabil. 2015 Jan 7;12(1):3. doi: 10.1186/1743-0003-12-3 (PMC4326518; doi:10.1186/1743-0003-12-3)

Additional file 1 Interaction plots of measurement results

RMS SEMG amplitude | mean standing/80% MVC

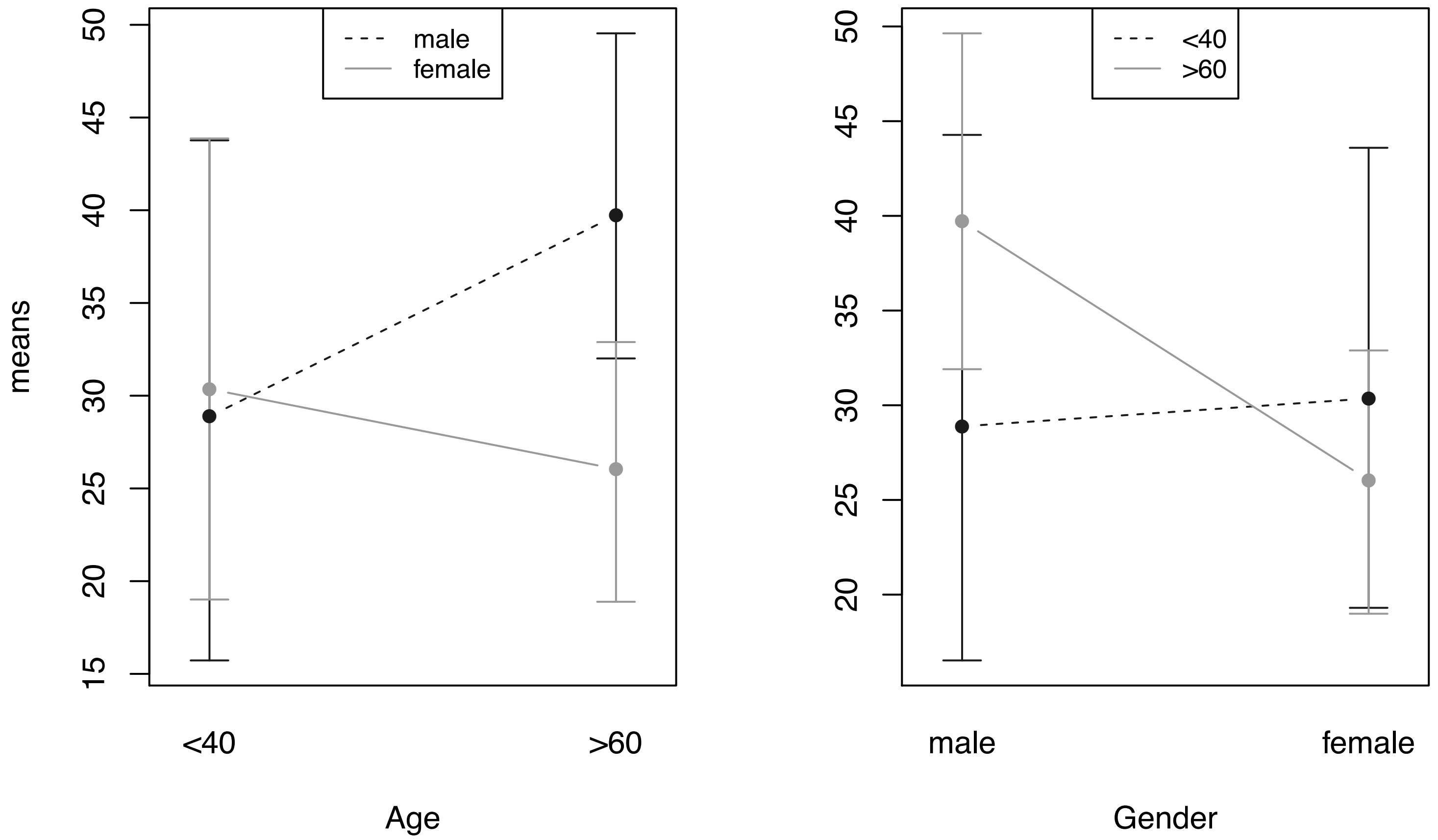

RMS SEMG amplitude | mean half flexion/80% MVC

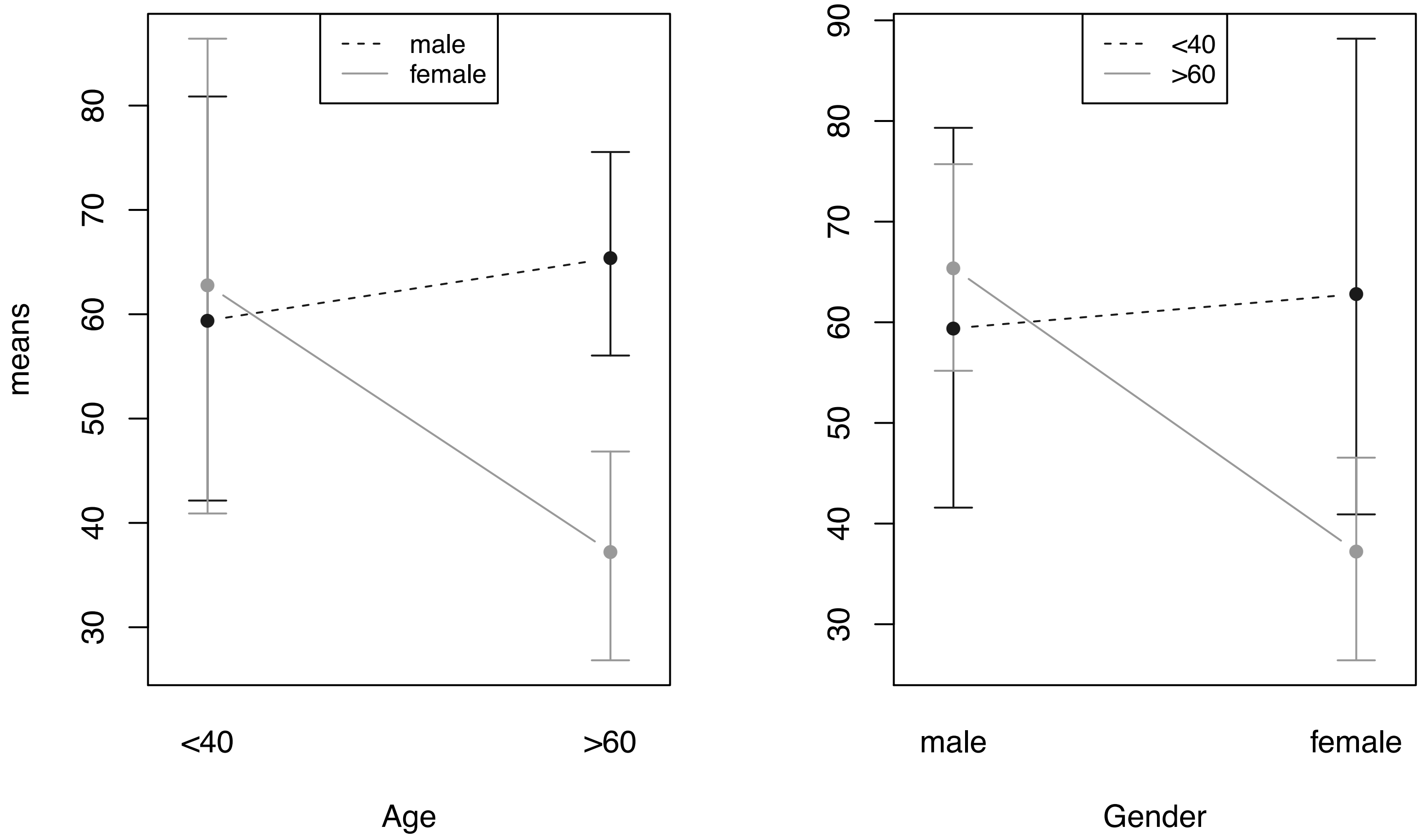

RMS SEMG amplitude | mean max flexion/80% MVC

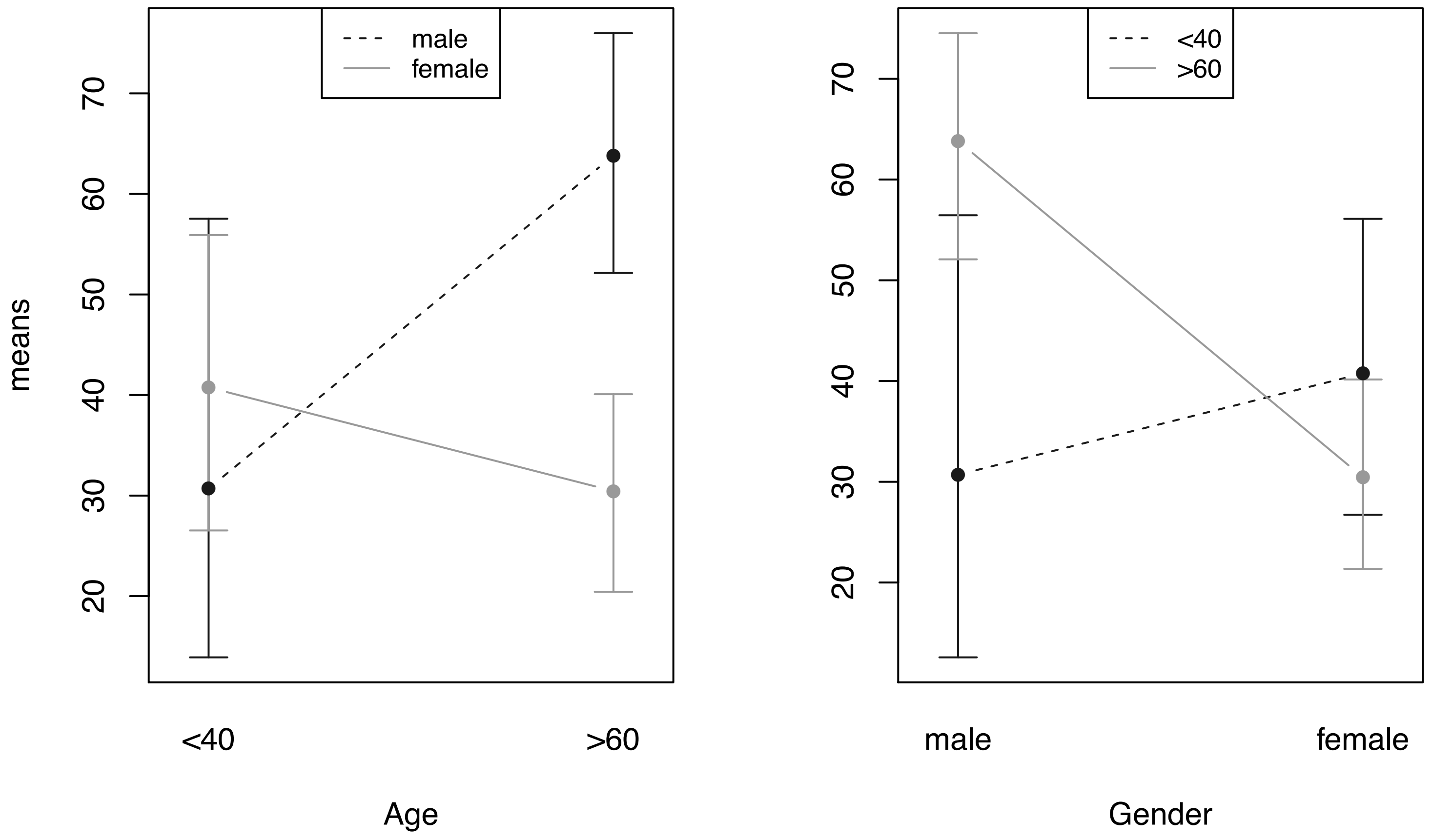

RMS SEMG changes | standing - half flexion

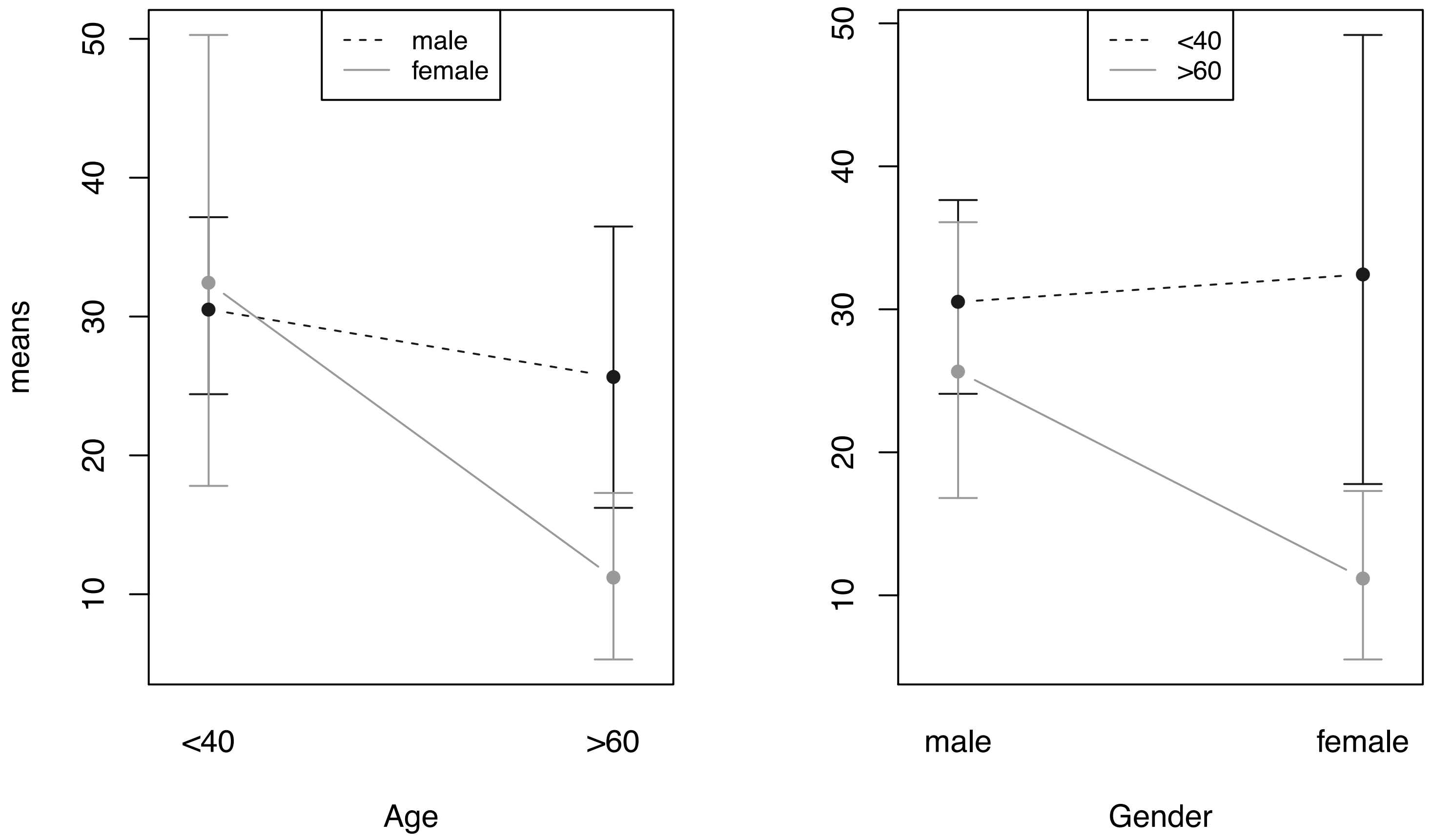

RMS SEMG changes | standing - max flexion

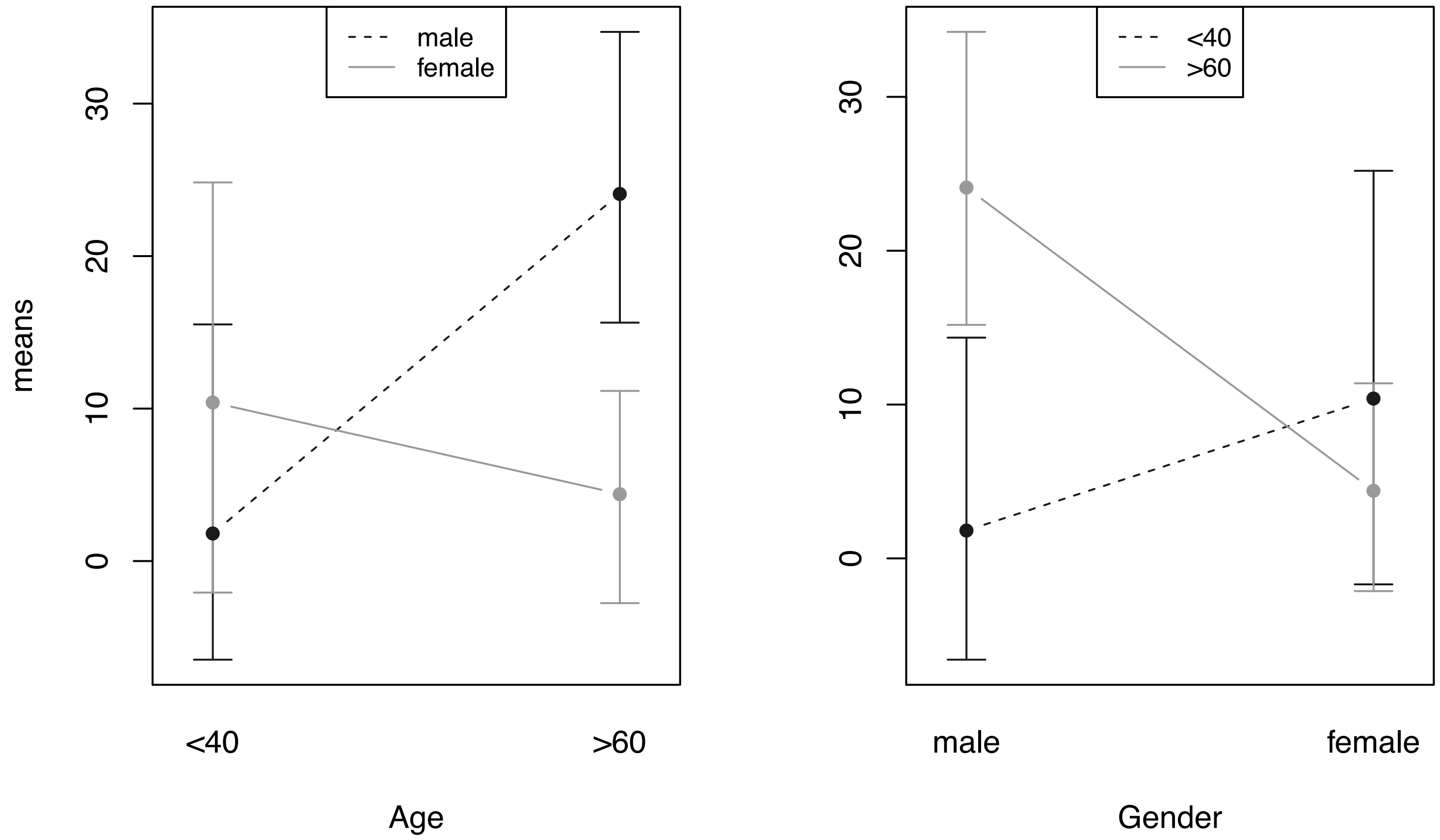

RMS SEMG changes | half flexion - max flexion

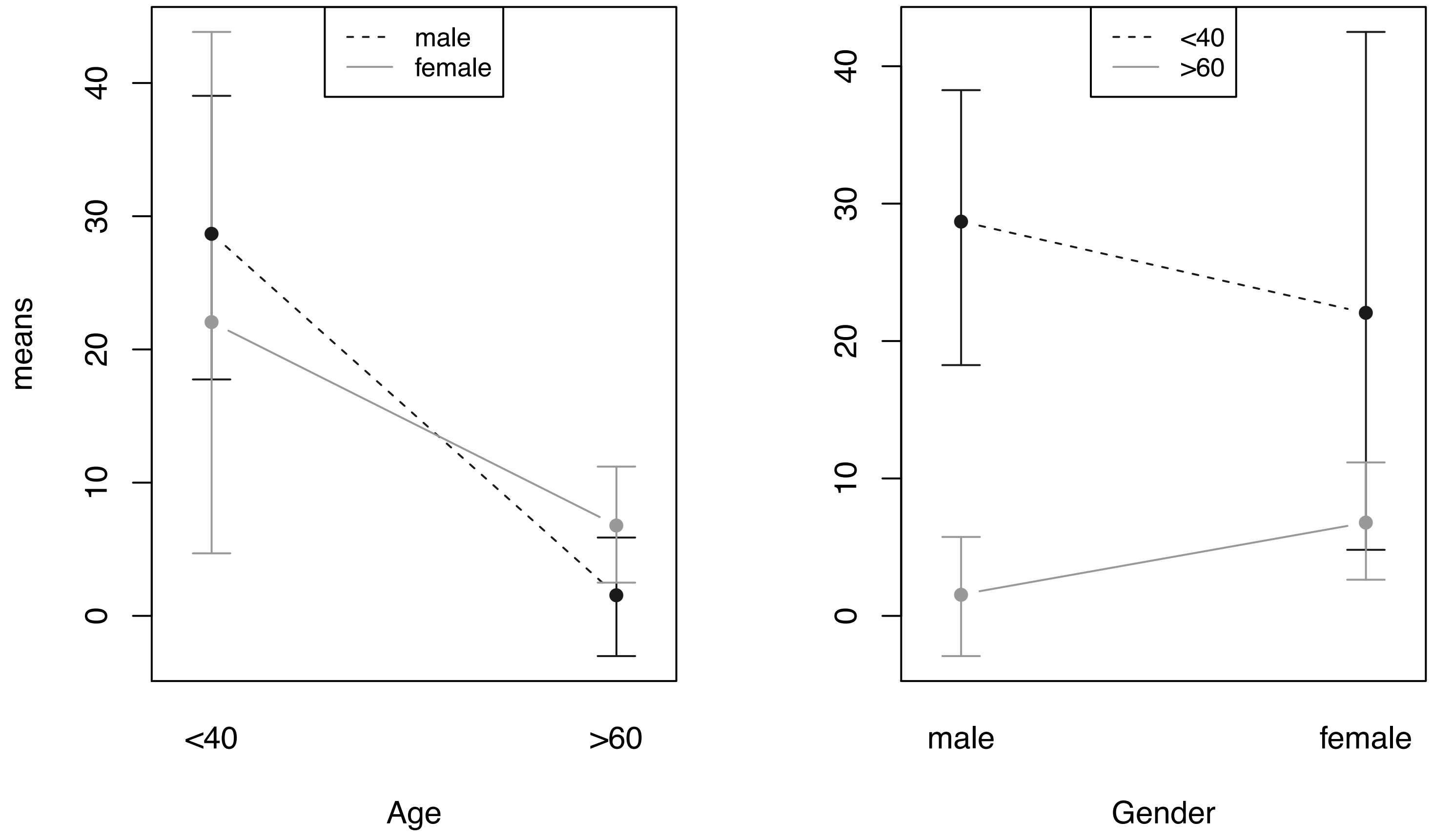

Flexion Relaxation Ratio (FRR) | mean half flexion/mean max flexion

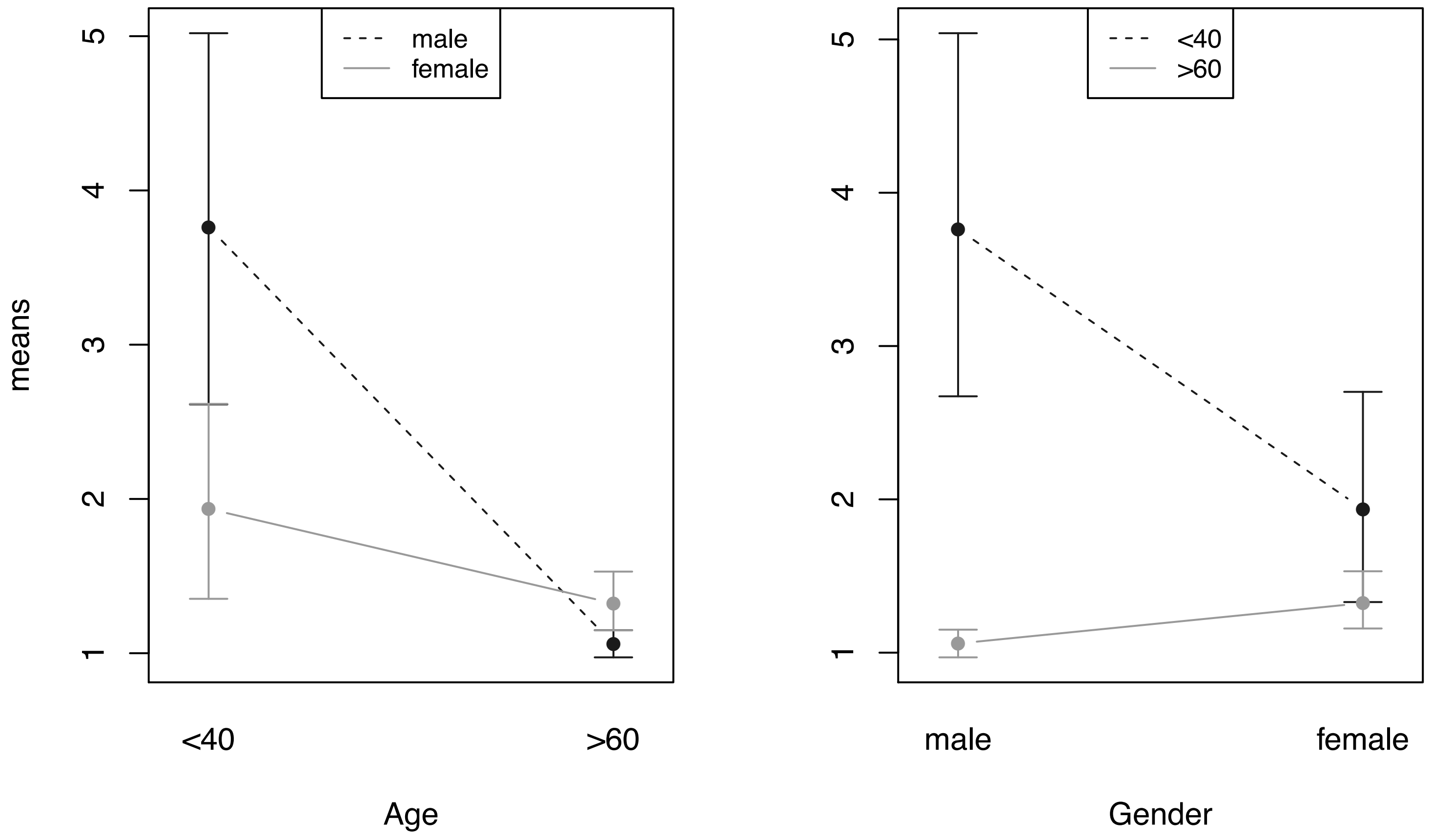

Range of Motion | hip | standing - max flexion

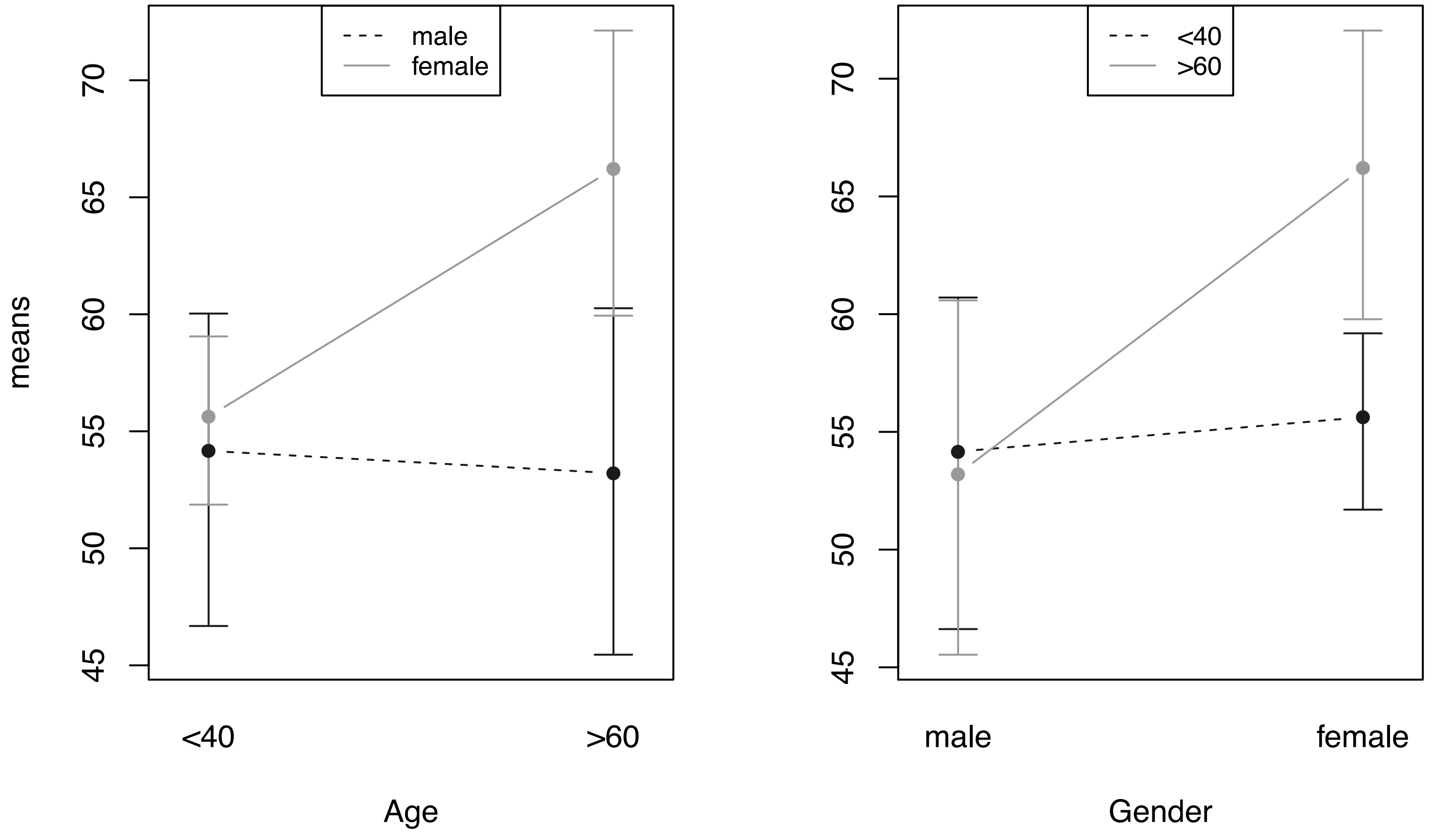

Range of Motion | lumbar | standing - max flexion

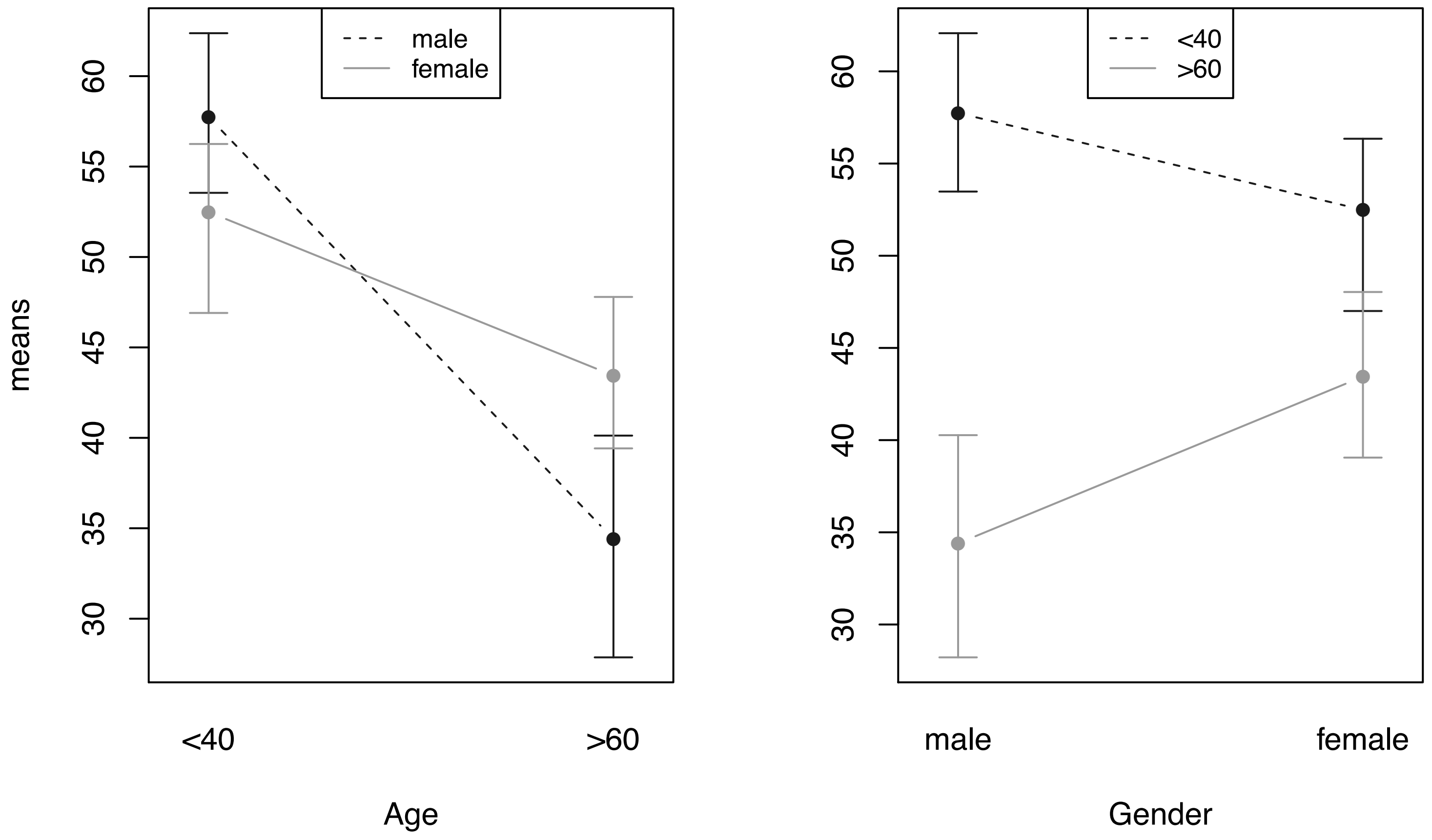

Range of Motion | gross trunk | standing - max flexion

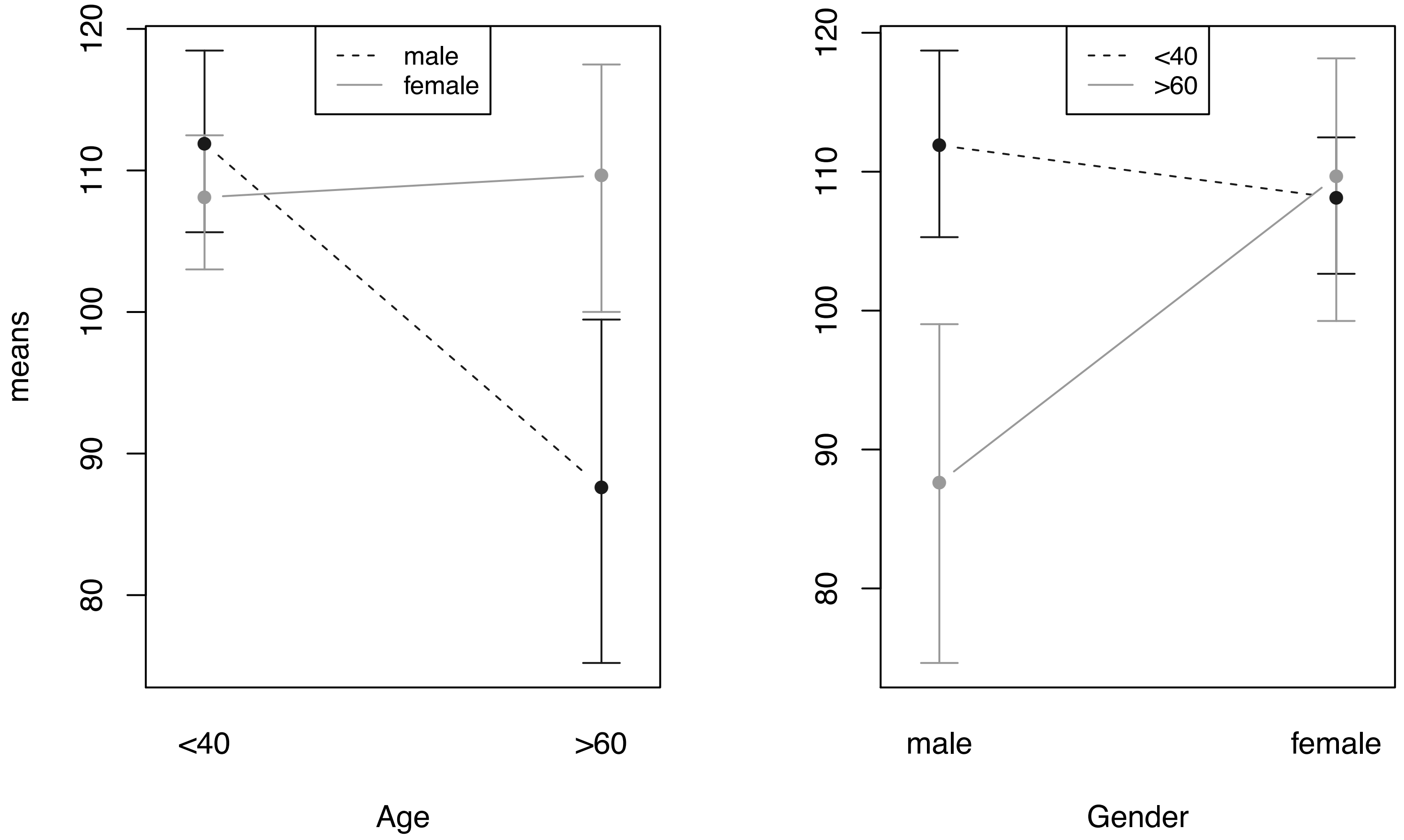

Range of Motion | hip | standing - half

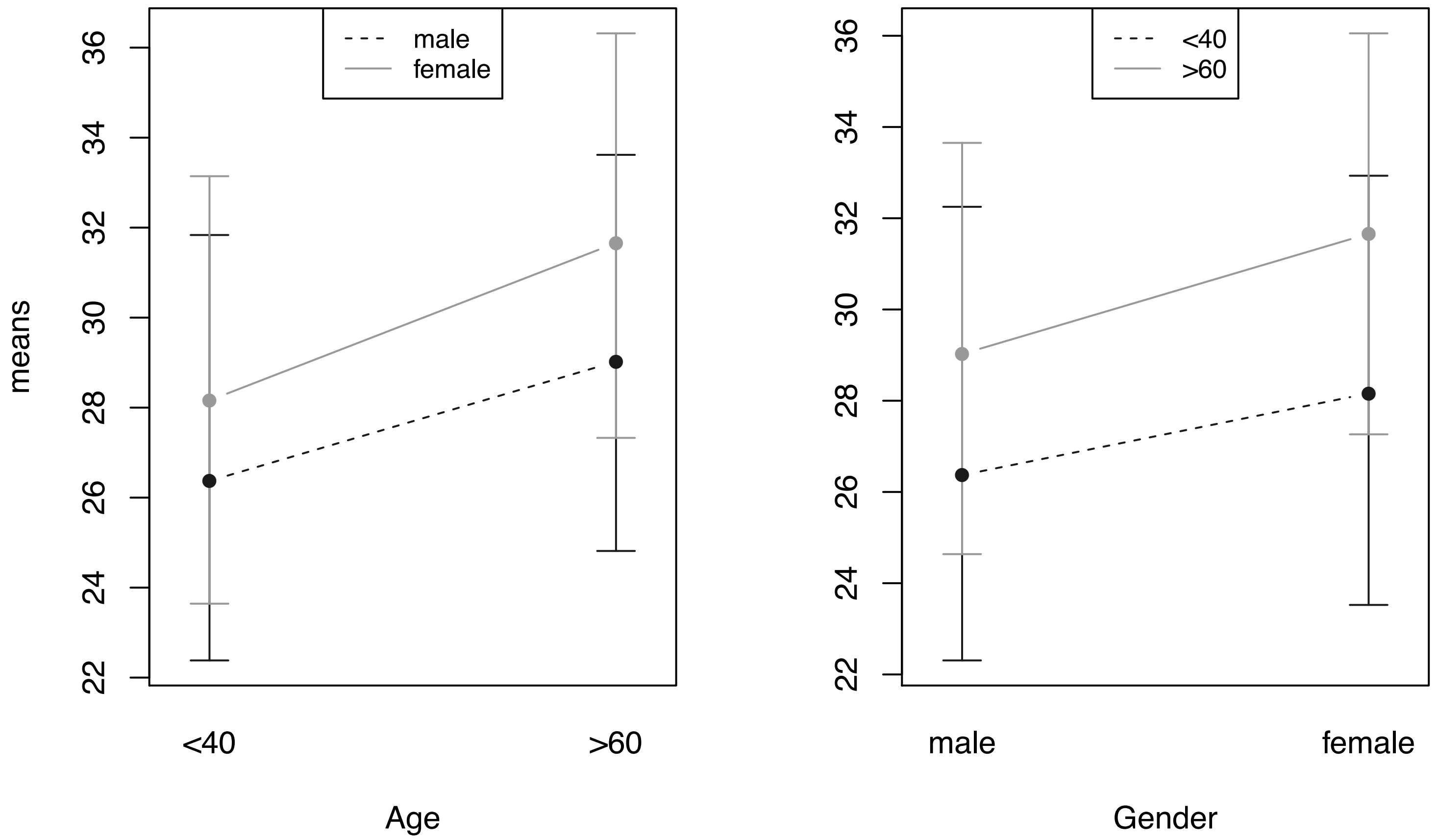

Range of Motion | lumbar | standing - half

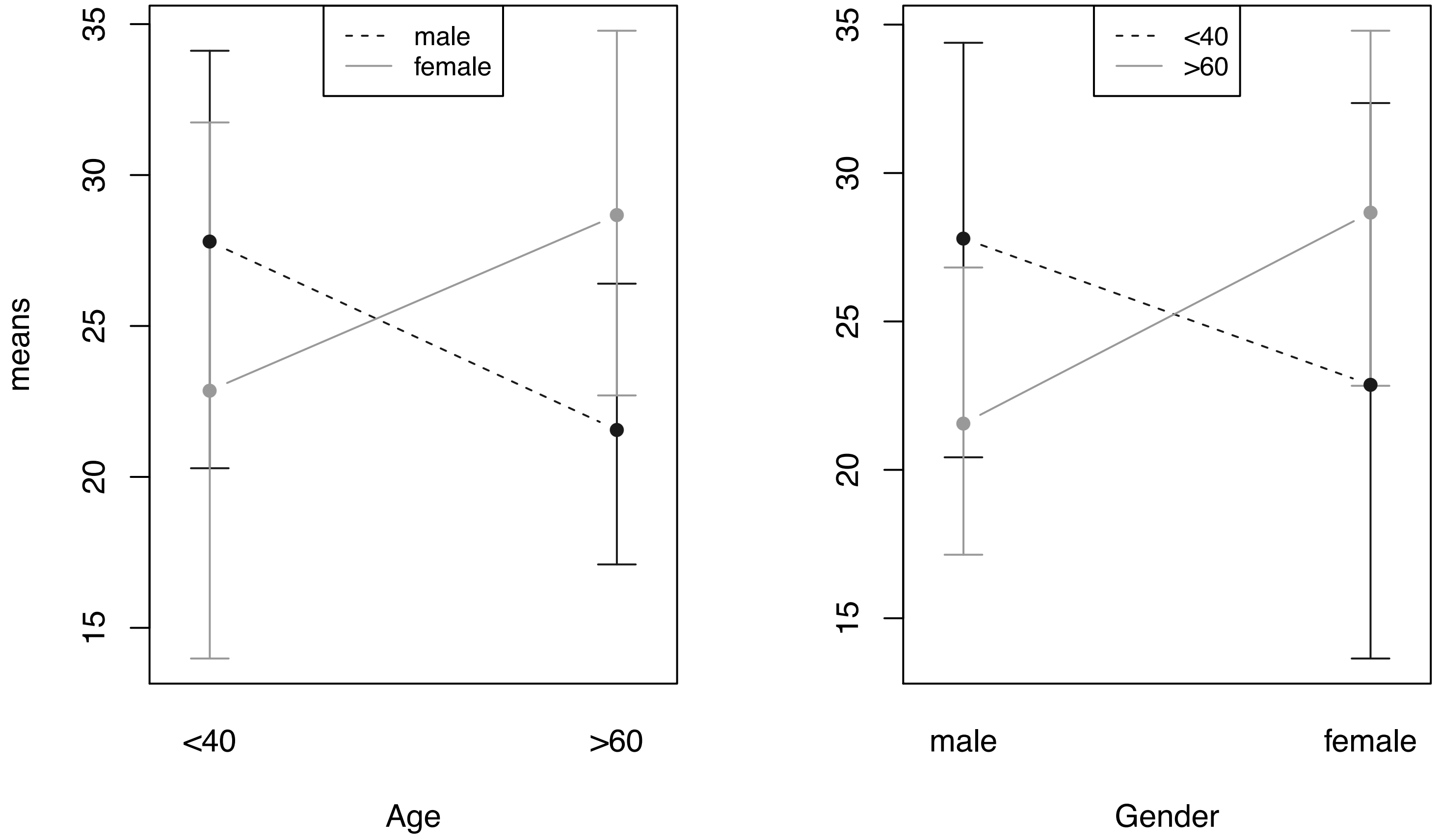

Range of Motion | gross trunk | standing - half

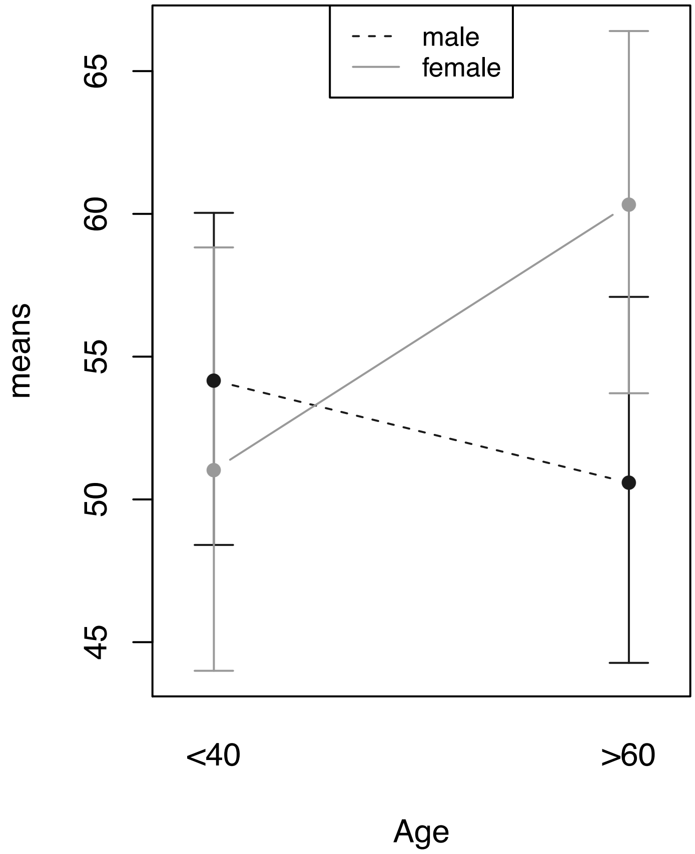

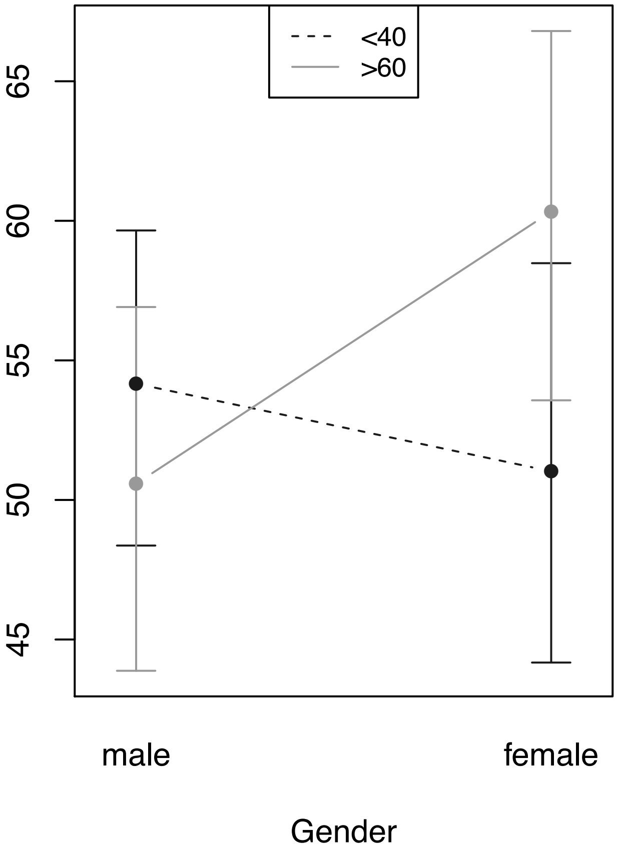

Supplement: Supplementary file 1 — Additional file 1: Interaction plots of measurement results. (PDF 210 KB) [file 12984_2014_696_MOESM1_ESM.pdf]
